# Radiomics analysis of R2* maps to predict early recurrence of single hepatocellular carcinoma after hepatectomy

**DOI:** 10.3389/fonc.2024.1277698

**Published:** 2024-02-23

**Authors:** Jia Li, Yunhui Ma, Chunyu Yang, Ganbin Qiu, Jingmu Chen, Xiaoliang Tan, Yue Zhao

**Affiliations:** ^1^ Department of Oncology, Central People’s Hospital of Zhanjiang, Zhanjiang, China; ^2^ Department of Radiology, The First School of Clinical Medicine, Shenzhen Maternity & Child Healthcare Hospital, Southern Medical University, Shenzhen, China; ^3^ Imaging Department of Zhaoqing Medical College, Zhaoqing, China; ^4^ Department of Radiology, Central People’s Hospital of Zhanjiang, Zhanjiang, China

**Keywords:** hepatocellular carcinoma, magnetic resonance imaging, early recurrence, radiomics analysis, nomogram

## Abstract

**Objectives:**

This study aimed to evaluate the effectiveness of radiomics analysis with R2* maps in predicting early recurrence (ER) in single hepatocellular carcinoma (HCC) following partial hepatectomy.

**Methods:**

We conducted a retrospective analysis involving 202 patients with surgically confirmed single HCC having undergone preoperative magnetic resonance imaging between 2018 and 2021 at two different institutions. 126 patients from Institution 1 were assigned to the training set, and 76 patients from Institution 2 were assigned to the validation set. A least absolute shrinkage and selection operator (LASSO) regularization was conducted to operate a logistic regression, then features were identified to construct a radiomic score (Rad-score). Uni- and multi-variable tests were used to assess the correlations of clinicopathological features and Rad-score with ER. We then established a combined model encompassing the optimal Rad-score and clinical-pathological risk factors. Additionally, we formulated and validated a predictive nomogram for predicting ER in HCC. The nomogram’s discrimination, calibration, and clinical utility were thoroughly evaluated.

**Results:**

Multivariable logistic regression revealed the Rad-score, microvascular invasion (MVI), and α fetoprotein (AFP) level > 400 ng/mL as significant independent predictors of ER in HCC. We constructed a nomogram based on these significant factors. The areas under the receiver operator characteristic curve of the nomogram and precision-recall curve were 0.901 and 0.753, respectively, with an F1 score of 0.831 in the training set. These values in the validation set were 0.827, 0.659, and 0.808.

**Conclusion:**

The nomogram that integrates the radiomic score, MVI, and AFP demonstrates high predictive efficacy for estimating the risk of ER in HCC. It facilitates personalized risk classification and therapeutic decision-making for HCC patients.

## 1 Introduction

Hepatocellular carcinoma (HCC) stands as the third leading cause of cancer-associated mortality globally (1). Partial hepatectomy with curative intent represents a pivotal strategy for early-stage HCC patients (2). Despite this, as many as 70% of HCC patients undergoing this therapy suffer recurrence within five years (1, 3). The timing of recurrence emerges as an independent survival factor, with early recurrence (ER) within two years correlating to lower overall survival (4). For these reasons, a risk stratification method to guide subsequent monitoring and treatment becomes imperative.

Previous studies (5–7) have identified several pathological factors, including microvascular invasion (MVI), vascular tumor thrombus, and histological grading, for HCC stratification. However, obtaining these pathological characteristics preoperatively through biopsy may not be possible in routine medical procedures due to potential bleeding risks. Furthermore, biopsy offers only a partial representation of HCC tissues, failing to capture the heterogenous characteristics of the entire mass. In contrast, imaging research may provide valuable insights into predicting postoperative ER in various malignancies. Magnetic resonance imaging (MRI), known for superior soft-tissue contrast and radiation-free imaging as an alternative to computed tomography (CT), has emerged as a non-invasive tool for detecting and characterizing HCC. MRI potentially provides biomarkers for predicting therapeutic responses and outcomes (8, 9). Certain traditional image features (e.g., non-smooth tumor margin, macrovascular vascular invasion, and peritumor hypointensity at the hepatobiliary phase [HBP]) are related to HCC outcomes (10–12). Despite the potential efficacy of these features, they remain limited and subjective (13, 14), presenting a challenge in terms of accurate prediction of ER.

The iterative decomposition of water and fat using echo asymmetry and least squares estimation (IDEAL IQ) creates an R2* map, which can quantify iron and reflect changes in oxygen content in local tissues (15). While the R2* map derived from IDEAL IQ has been utilized to assess iron content relevant to certain liver diseases, such as iron overload and fibrosis (16), its application to determine ER in HCC after hepatectomy is yet unexplored. Given that malignant HCC elevates blood metabolite levels due to increased oxygen consumption from active tumor cell proliferation, we hypothesized that elevated R2* values could serve as a predictive factor for ER in HCC.

Radiomics, an emerging field, involves the extraction of high-dimensional, mineable, quantitative features from medical imaging breaking through the limitations of visual assessment. In HCC, radiomic models have shown potential applications in predicting histology, treatment response, recurrence, and survival. In the present study, we aimed to develop a radiomics model based on a preoperative R2* map to predict ER in HCC patients after hepatectomy. Additionally, we sought to create and test a combined nomogram for ER prediction, integrating the radiomic score derived from the optimal performing model with clinicopathologic-radiologic variables. This approach is designed to stratify HCC patients, thereby improving the outcomes of personalized treatment.

## 2 Materials and methods

### 2.1 Population and follow-up approaches

**Figure 1:**
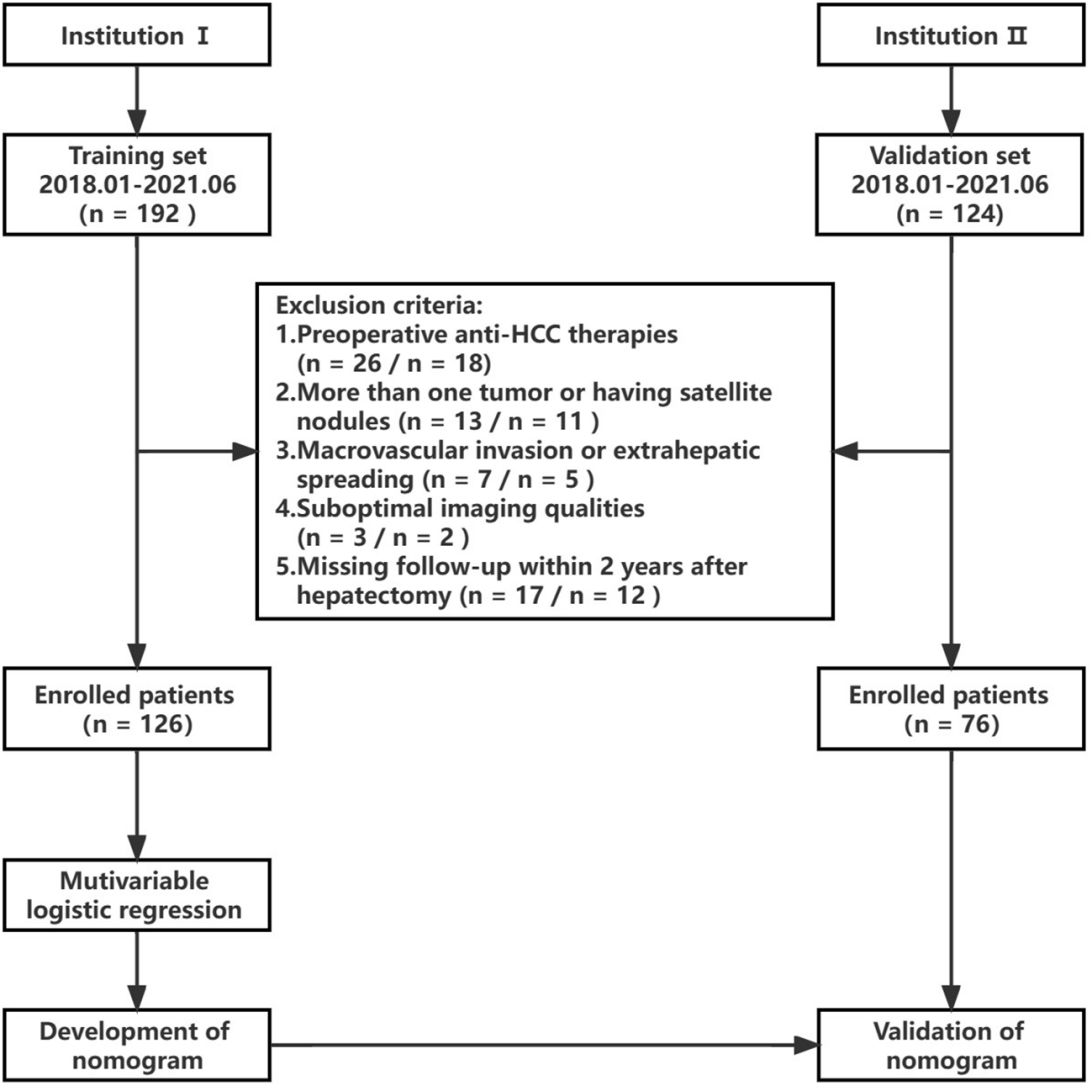
Study flowchart.

This study received approval from the institutional ethics review board. The need for informed consent was waived due to the retrospective nature of the investigation. Data were gathered from patients at Institution 1 and Institution 2 spanning 2018 to 2021. **Figure 1** shows the flowchart of this study’s design. The inclusion criteria included individuals 1) with pathologically confirmed single HCCs, 2) aged ≥ 18 years, 3) having undergone enhanced MRI no more than two weeks prior to surgery, and 4) possessing complete clinicopathologic data. Exclusion criteria encompassed those 1) opting for alternative therapies like radiofrequency ablation or transcatheter arterial chemoembolization (TACE) rather than resection surgery, 2) presenting with satellite nodules or more than one tumor, 3) exhibiting extrahepatic spreading or macrovascular invasion, 4) having inadequate image quality for interpretation, and 5) lacking follow-up within two years post-hepatectomy. The training set for modeling to predict ER in HCC comprised 126 patients from Institution 1, while the validation set included 76 HCC patients from Institution 2.

Regular monitoring for recurrence in all HCC patients involved contrast CT or MRI each three months for two years post-resection, with a follow-up deadline set at June 2023. Recurrence criteria were defined as the emergence of extrahepatic metastasis or new intrahepatic lesions. These criteria included new intrahepatic lesions displaying typical HCC imaging features, confirmed by tumor staining during postoperative TACE, or histopathology, as well as extrahepatic metastasis verified through typical imaging features or histopathological assays.

### 2.2 Clinical and pathological data

Clinical data were collected including age, sex, hepatitis B viral infection status, and various laboratory indices, such as α-fetoprotein (AFP), alanine aminotransferase, aspartate aminotransferase, glutamyl transpeptidase, serum creatinine, alkaline phosphatase, total and direct bilirubin, prothrombin time, albumin, platelet-to-lymphocyte ratio (PLR), and neutrophils-to-lymphocyte ratio (NLR).

Two pathologists, each possessing over eight years of HCC pathology-related experience, independently examined all sample slices without access to the clinical data. In cases of disagreement, we consulted a third senior pathologist (with 20 years of experience) to provide resolution. MVI was defined as the presence of tumor cell clusters inside a vascular space of the peripherical hepatic tissue lined by endothelium, visible only under microscopic examination (17). The histological division was determined using the Edmondson and Steiner (E-S) grade. In instances where multiple tumor grades coexisted, the highest grade was utilized for diagnosis. E-S grades 1 and 2 imply high differentiation, while grade 3 and grade 4 denote low differentiation. The Ki-67 labeling index (LI) was assessed by computing the proportion of Ki-67-positive cells. Positive Ki-67 was identified if the nuclei were stained brownish yellow. Low and high Ki-67 LI were classified by immunoreactive cells with ≤10% and > 10% immune reactivity, respectively (18).

### 2.3 MRI protocol

A uniform MRI scanner and scanning protocol were applied for all patients across both institutions. The MRI procedures were conducted using a 3.0 T system (Discovery 750w, GE Healthcare). Standard liver protocols included axial breath-hold IDEAL IQ, axial T2-weighted fast spin-echo sequence, and axial breath-hold T1-weighted three-dimension fat-suppressed spoiled gradient-echo sequence with liver acquisition and volume acceleration. Following this, Gd-diethylenetriamine pentaacetic acid (Gd-DTPA, Bayer Schering Pharma, Germany) contrast agent was administered via the cubital vein at 1.0 ml/s and 0.025 mmol/kg. Subsequently, the T1-weighted three-dimension fat-suppressed spoiled gradient-echo sequence was repeated. The dynamic contrast-enhanced scanning process included arterial phase (AP, 20-45 s), portal vein phase (PVP, 50-75 s), and delayed phase (DP, 90 s) images. **Table 1** displays detailed parameters for each sequence.

**Table 1:** MR imaging sequence parameters.

Parameters	Axial breath-hold iterative decomposition of water and fat with echo asymmetry and least squares estimation (IDEAL IQ)	Axial breath-hold T1-weighted three-dimension fat suppressed spoiled gradient-echo sequence with liver acquisition and volume acceleration	Axial T2-weighted fast spin-echo	Contrast enhanced T1-weighted imaging
Echo time (ms)	1.0	1.5	72.5	1.45
Repetition time (ms)	6.5	4.0	4255	3.27
Field of view (mm^2^)	400×400	380×380	360×360	380×380
Bandwidth (Hz)	1322	762	320	762
Section thickness (mm)	5	5	5	5
Flip angle (degree)	3	12	120	10

### 2.4 Radiomics analysis

**Figure 2:**
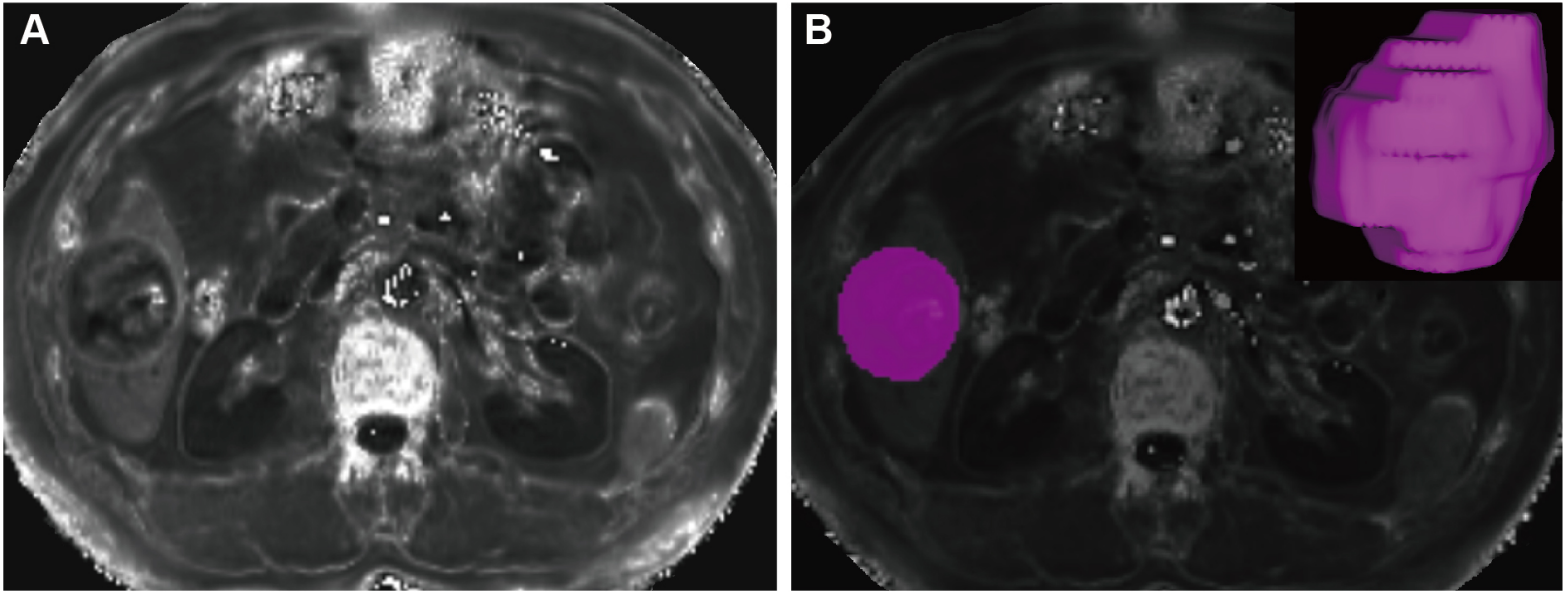
Tumor segmentation. A mass located in the hepatic segment VI with hyperintense in the R2* maps **(A)**. The tumor was segmented on R2* maps and the corresponding volume-rendering image **(B)**.

All images were imported to ITK-SNAP (3.4.0 version, http://www.itksnap.org/) for segmentation. A pair of radiologists, both with seven years of abdominal diagnosis experience, performed the segmentation blindly to clinicopathologic data and follow-up information. Each manually delineated the region of interest (ROI) layer-by-layer, ensuring the accuracy of segmentation on R2* maps by carefully referencing enhanced MRI images for determination of the ROI edge. Subsequently, the software automatically generated the three-dimensional ROI of the entire lesion (**Figure 2**).

All segmented data were then transferred to A.K. (Artificial Intelligence Kit 3.0.1. A, GE Healthcare). Radiomic features were extracted using Pyradiomics, an open-source Python package. Inter-radiologist agreement was assessed by intra-class correlation (ICC). Only the features with ICC > 0.75 were included in the candidate feature set. This set ultimately comprised a total of 91 texture features (ICC = 0.787-0.931), including 1) 16 first-order features; 2) 24 gray-level co-occurrence matrix (GLCM) features; 3) 14 gray-level dependence matrix (GLDM) features; 4) 16 gray-level size zone matrix (GLSZM) features; 5) 16 gray-level run length matrix (GLRLM) features; and 6) five neighboring gray-tone difference matrix (NGTDM) features.

The training set was subjected to feature selection and modeling, followed by verification on the validation set. The extracted radiomic features were optimized via general correlation analysis, univariable analysis, and least absolute shrinkage selection operator (LASSO) regression. A logistic regression (LR) classifier was employed for machine learning to build a predictive model, forming a linear weighted amalgamation of the optimal features and their coefficients. This combination was utilized to calculate the radiomics score (Rad-score). The model performance was estimated using five-fold cross-validation. In each cross-validation iteration, the optimal features were evaluated by the feature selection and then transferred to the LR classifier and modeled. To determine the predictive error and confidence interval for training and validation sets, we operated the model via 1000-iteration bootstrap analysis for both. We randomly chose a subset of 75% of cases for each repetition from one of the sets.

### 2.5 Statistical analysis

Continuous data were recorded as mean and standard deviation or as median with interquartile values, while categorical data were expressed as numbers and proportions. Data-distribution normality was calculated with a Shapiro-Wilk test. Group comparisons involved a Student’s t-test and Mann-Whitney U-test for normally and non-normally distributed continuous data, respectively. Binary categorical data were evaluated with chi-square tests.

We generated receiver operating characteristic (ROC) curves to test the model’s classification performance for both sets of data. Model performance metrics, including accuracy, sensitivity, specificity, negative predictive value (NPV), positive predictive value (PPV), and the area under the curve (AUC) were determined. Model fit was examined using calibration curves and a Hosmer-Lemeshow (H-L) test. The clinical benefit of the model was evaluated according to clinical decision and impact curves. All data analysis was performed in R software (R Studio 3.4.4, https://www.r-project.org), with *P* < 0.05 denoting statistical significance.

## 3 Results

### 3.1 Clinicopathological features of the training and validation sets

We considered 202 patients, including 126 from institution I (41 ER patients, 85 non-ER patients) and 76 from Institution 2 (23 ER patients and 53 non-ER patients). Their baseline clinical and pathological information are displayed in **Table 2**. In the training set, inter-group AFP, NLR, PLR, MVI, E-S grade, and Ki67 LI significantly differed (all *P* < 0.05). However, the distribution of clinicopathological data was similar between the two groups (all *P* > 0.05).

**Table 2:** Baseline clinical and pathological characteristics of the training and validation sets.

Characteristics	Training set (n = 126)				Validation set (n = 76)	*p*_Inter_
	Total (n = 126)	ER (n = 41)	Non-ER (n = 85)	*p*_Intra_		
Age (years)	63 (52 ~ 68)	65 (54 ~ 72)	59 (51 ~ 67)	0.258	52 (43 ~ 65)	0.286
Sex (male)	115 (91.3)	38 (92.7)	77 (90.6)	0.696	65 (85.5)	0.632
HBsAg				0.309		0.176
Negative	25 (19.8)	6 (14.6)	19 (22.4)		14 (18.4)	
Positive	101 (80.2)	35 (85.4)	66 (77.6)		62 (81.6)	
ALT (U/L)	37.00 (22.00 ~ 65.25)	40.00 (27.00 ~ 69.00)	34.00 (20.00 ~ 56.50)	0.143	31.50 (20.60 ~ 54.50)	0.653
AST (U/L)	41.00 (26.00 ~ 49.00)	42.00 (30.00 ~ 54.50)	37.00 (23.50 ~ 58.50)	0.174	41.50 (23.00 ~ 54.75)	0.836
GGT (U/L)	67.00 (42.00 ~ 139.75)	81.00 (42.00 ~ 147.00)	63.00 (42.50 ~ 129.00)	0.438	55.50 (36.75 ~ 126.75)	0.653
ALP (U/L)	83.00 (68.75 ~ 104.25)	86.00 (71.50 ~ 116.00)	80.00 (65.00 ~ 99.50)	0.449	88.50 (69.00 ~ 108.75)	0.557
ALB (g/L)	40.50 (36.78 ~ 43.00)	41.00 (36.65 ~ 43.15)	40.00 (36.95 ~ 42.90)	0.603	39.50 (36.70 ~ 42.70)	0.162
TBIL (µmol/L)	14.87 (11.39 ~ 18.55)	14.99 (12.12 ~ 19.59)	14.80 (10.95 ~ 17.75)	0.246	14.50 (10.60 ~ 18.78)	0.667
DBIL (µmol/L)	4.94 (2.90 ~ 7.18)	4.62 (3.03 ~ 5.94)	5.36 (2.88 ~ 7.63)	0.606	3.45 (2.40 ~ 5.28)	0.167
SCr (U/L)	74.50 (66.93 ~ 86.85)	73.20 (63.00 ~ 82.15)	76.00 (67.00 ~ 87.50)	0.126	74.50 (66.28 ~ 88.30)	0.648
PT (s)	11.90 (11.37 ~ 12.53)	12.90 (11.50 ~ 12.60)	11.90 (11.20 ~ 12.45)	0.323	11.75 (10.40 ~ 14.58)	0.456
NLR	1.98 (1.51 ~ 3.02)	2.44 (1.60 ~ 3.79)	1.88 (1.48 ~ 2.62)	0.007*	2.03 (1.55 ~ 3.62)	0.543
PLR	107.91 (70.45 ~ 149.20)	117.14 (75.35 ~ 184.00)	102.56 (65.23 ~ 141.29)	0.035*	106.87 (76.30 ~ 156.82)	0.875
AFP (ng/mL)				<0.001*		0.653
≤400	89 (70.6)	21 (51.2)	68 (80.0)		56 (73.7)	
>400	37 (29.4)	20 (48.8)	17 (20.0)		20 (26.3)	
Microvascular invasion				<0.001*		0.464
Absent	81 (64.3)	16 (39.0)	65 (76.5)		45 (59.2)	
Present	45 (35.7)	25 (61.0)	20 (23.5)		31 (40.8)	
Edmondson-Steiner grade				0.004		0.338
I-II	75 (59.5)	17 (41.5)	58 (68.2)		40 (52.6)	
III-IV	51 (40.5)	24 (58.5)	27 (31.8)		36 (47.4)	
Ki-67 labeling index				0.013*		0.326
≤10%	47 (37.3)	9 (22.0)	38 (44.7)		28 (36.8)	
>10%	79 (62.7)	32 (78.0)	47 (55.3)		48 (63.2)	

Continuous variables are presented as median (inter-quartile range, IQR). The categorical variables are presented as numbers (percentages). Using univariable association analyses, *P*_Intra_ is the result of univariate analyses between ER and no ER groups, while *P*_Intra_ represents whether a significant difference exists between the training and validation datasets.

HBsAg, hepatitis B surface antigen; ALT, alanine aminotransferase; AST, aspartate aminotransferase; GGT, glutamyl transpeptidase; ALP, alkaline phosphatase; ALB, albumin; TBIL, total bilirubin; DBIL, direct bilirubin; SCr, serum creatinine; PT, prothrombin time; NLR, neutrophil to lymphocyte ratio; PLR, platelet to lymphocyte ratio; AFP, alpha-fetoprotein. *P<0.05.

### 3.2 Feature selection and rad-score

Following univariable and correlation analysis in the training set, 24 of the candidate features were retained. Subsequent LASSO regression and cross-validation further narrowed down the features to six, including two first-order features (maximum, median), one GLRLM feature (run entropy [RE]), one GLDM feature (dependence variance [DV]), and two GLSZM features: gray-level variance (GLV) and gray-level non-uniformity (GLN) (**Table 3**).

**Table 3:** Comparison of feature values between ER group and non-ER group in training set and validation set.

Feature	Training set			Validation set		
	ER group (n = 41)	Non-ER group (n = 85)	*P*	ER group (n = 23)	Non-ER group (n = 53)	*P*
First order						
Maximum	60.00 (65.40, 71.60)	52.00 (48.70, 66.00)	<0.001	58.00 (62.31, 68.45)	53.25 (44.34, 67.54)	<0.001
Median	46.45 ± 12.74	34.48 ± 11.20	<0.001	43.32 ± 10.64	32.45 ± 11.43	<0.001
GLDM						
DV	34.12 ± 10.67	23.65 ± 10.85	<0.001	36.56 ± 8.70	18.26 ± 9.45	<0.001
GLRLM						
RE	3.28 ± 0.36	2.75 ± 0.34	<0.001	3.45 ± 0.54	2.78 ± 0.43	
GLSZM						
GLN	78.50 (43.60, 145.47)	28.12 (11.76, 43.88)	<0.001	82.59 (63.76, 150.54)	34.54 (8.17, 25.47)	<0.001
GLV	0.96 (0.89, 1.15)	0.86 (0.41, 1.10)	0.004	1.15 ± 0.62	0.82 ± 0.42	0.028

The Mann-Whitney *U* test was applied in the analysis of maximum, GLN, and GLV comparison, the independent sample t test was applied in the rest of the comparisons. Data of median, DV, and RE are means ± standard deviations, the rest of data are described as medians (quartiles).

ER, ER; GLDM, gray-level dependence matrix; GLRLM, gray-level run length matrix; GLSZM, gray-level size zone matrix; DV, dependence variance; RE, run entropy; GLN, gray-level non-uniformity; GLV, gray-level variance.

The six optimal texture features were compared to find that the ER group feature values had higher values than the non-ER group across both training and test sets (**Table 3**). The linear combination of the weighted coefficients for six features formed the following Rad-score equation: Rad-score = -0.38 + 1.02 median - 1.45 maximum + 0.86 RE + 1.15 DV - 0.45 GLN + 1.56 GLV.

### 3.3 Performance of prediction models

Multivariable analysis revealed independent significant indices for ER in HCC as serum AFP level > 400 ng/mL (OR [95% CI] = 5.721 [1.585 - 20.649], *P* = 0.008), MVI (4.854 [1.404 - 16.782], *P* = 0.013) and Rad-score (3.352 [2.008 - 5.597], *P* < 0.001) (**Table 4**). The H-L test yielded a significance level of 0.392, indicating the model’s acceptable goodness-of-fit.

**Table 4:** Univariable and multivariable logistic regression of clinical and texture features for ER in HCC.

Characteristics	Univariable		Multivariable	
	OR (95% CI)	*P* value	OR (95% CI)	*P* value
AFP > 400 ng/mL	8.317 (3.576 ~ 19.343)	<0.001	5.721 (1.585 ~ 20.649)	0.008
NLR	1.738 (1.197 ~ 2.523)	0.004		
PLR	1.005 (1.000 ~ 1.010)	0.040		
Rad-score	6.777 (2.309 ~ 19.891)	<0.001	3.352 (2.008 ~ 5.597)	<0.001
MVI	12.727 (5.238 ~ 30.925)	<0.001	4.854 (1.404 ~ 16.782)	0.013
Edmondson-Steiner grade	3.033 (1.403 ~ 6.557)	0.005		
Ki-67 labeling index	2.875 (1.224 ~ 6.754)	0.015		

OR, odds ratio; CI, confident interval; AFP, alpha-fetoprotein; NLR, neutrophil to lymphocyte ratio; PLR, platelet to lymphocyte ratio; MVI, microvascular invasion.

**Figure 3:**
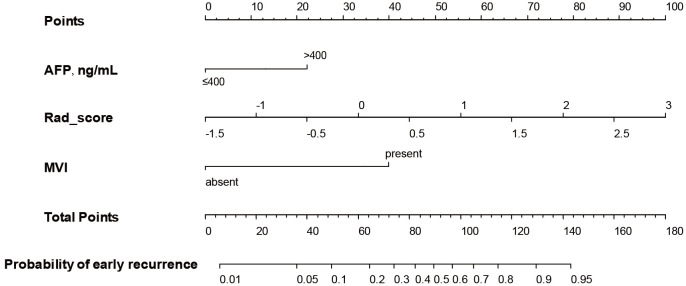
The nomogram was developed based on radiomics score, MVI, and serum AFP level.

**Figure 4:**
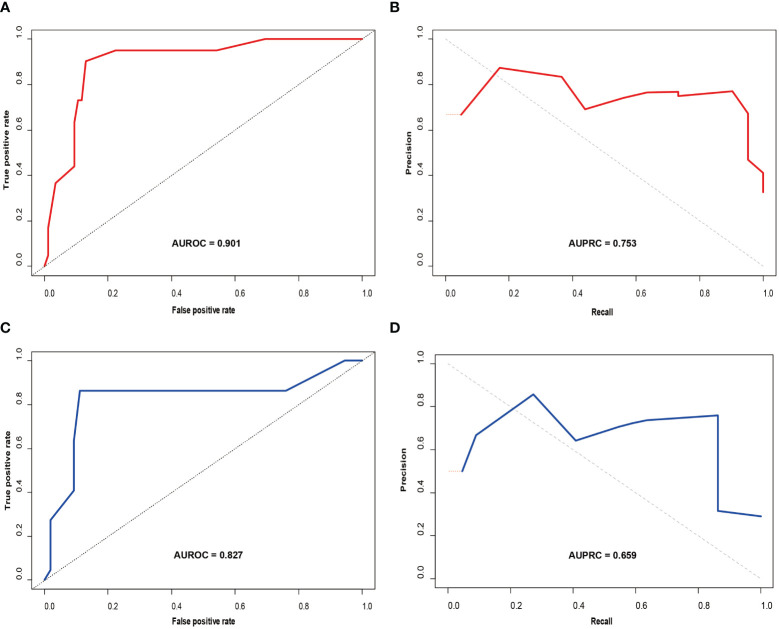
Receiver operating characteristic (ROC) curve **(A)** and precision-recall (PR) curve **(B)** for the prediction model in the training set; ROC curve **(C)** and PR curve **(D)** in the validation set.

A nomogram (**Figure 3**) for forecasting ER in HCC was constructed based on AFP and Rad-score values, exhibiting an AUC of 0.901 (95% CI: 0.826-0.947). The corresponding sensitivity, accuracy, specificity, NPV, and PPV were 90.2%, 88.1%, 87.1%, 90.8%, and 77.1%, respectively. Precision-recall analysis demonstrated an AUC of 0.753 and an F1 score of 0.831. For the external validation dataset, these indices were 0.827 (0.701-0.924), 86.4%, 88.2%, 88.9%, 76.0%, 94.1%, 0.808, and 0.659, respectively. ROC and precision-recall curves of the nomogram for the training and validation sets are illustrated in **Figure 4**.

**Figure 5:**
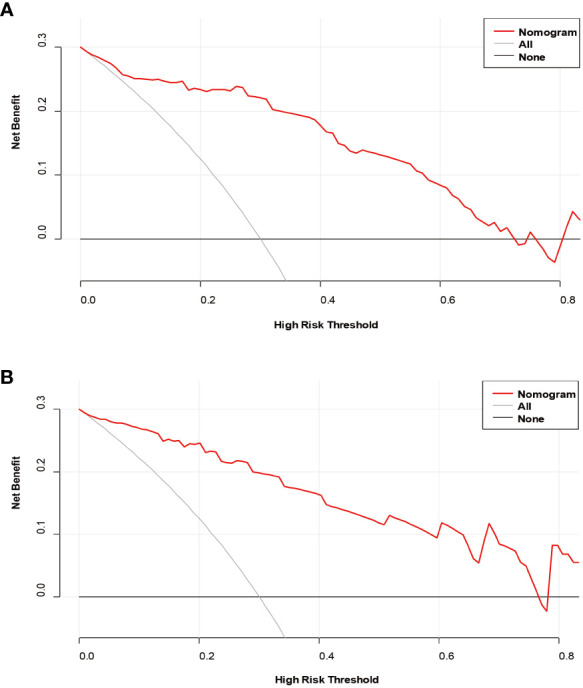
Decision curve analysis of the prediction model in the training **(A)** and validation **(B)** sets.

**Figure 6:**
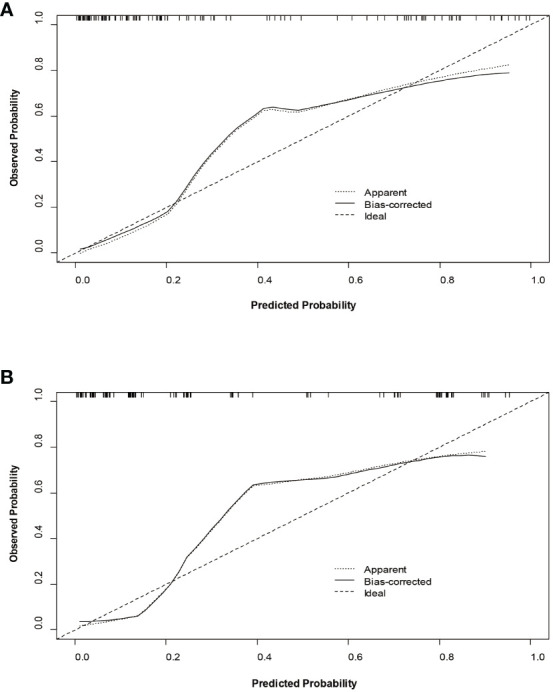
Calibration curves of the nomogram in the training set **(A)** and validation set **(B)**.

The decision curves (**Figure 5**) revealed that the model had significant clinical benefits for predicting ER in HCC. Calibration curves (**Figure 6**) exhibited consistent predicted and observed likelihood of ER for the training set (*P* = 0.265) and validation set (*P* = 0.569).

## 4 Discussion

Current guidelines suggest surgical hepatectomy as the primary treatment for HCC patients, particularly those with solitary HCC. Despite this, the high postoperative recurrence rate remains a challenge, and the absence of a reliable prediction tool is problematic (1, 2). In this study, we retrospectively examined R2* maps from 126 single HCC patients, verified by postoperative pathology, utilizing texture analysis to derive six optimal texture features and compute Rad-scores. Subsequently, we established and evaluated a nomogram based on Rad-scores, MVI, and serum AFP levels to predict the ER of HCC patients. The results indicate that the proposed model holds potential for assisting HCC patients with individualized risk classification and guiding therapeutic decision-making.

Traditional quantitative parameters involve manually drawing ROIs, introducing subjective factors. Variability in ROI drawing positions and selecting a single slice or several slices can impact the results and cause the heterogeneity of the entire tumor to be neglected (19, 20). Utilizing radiomics analysis to outline the entire tumor and obtain radiomic parameters offers a more objective and comprehensive reflection of the tumor’s heterogeneity. First-order features can reveal histogram characteristics across all voxels, while GLCM features reflect gray-level distribution characteristics and the positional distribution between pixels with similar gray levels. GLSZM, GLDM, and GLRLM features quantify the regions of continuous pixel values, grayscale dependency, and the distribution of pixel values, respectively. Moreover, the NGTDM feature quantifies the difference between a given grayscale value and the average grayscale value within an adjacent distance (19, 21). In this study, six optimal texture features were determined to describe tumor uniformity, including DV (GLDM), RE (GLRLM), GLN, and GLV (GLSZM). We hypothesize that this may be attributed to actively proliferating HCC cells prone to ER and increased abnormal neovascularization in the tumor. This disorganized neovascularization, often associated with ruptured duct walls, heightened susceptibility to hemorrhage and necrosis, and pronounced tumor anisotropy, may contribute to greater heterogeneity in signal intensity within the tumor (22). The maximum and median intensity values, as measured on the R2* maps, differed between the two groups evaluated in this study. Specifically, the ER group displayed higher signal intensity. HCCs prone to ER exhibit higher malignancy and more active proliferation of tumor cells; the tumor consumes increased levels of oxygen, resulting in elevated levels of paramagnetic substances like blood metabolites (e.g., ferritin and deoxyhemoglobin). Consequently, the R2* values increase (23). The Rad-score computed in this study comprises specific categories, including two histogram-based features (maximum and median), one GLDM feature (DV), one GLRLM feature (RE), and two GLDM features (GLN and GLV). This Rad-score accurately and comprehensively reflects tumor biology and heterogeneity by reflecting the layout of pixel intensity within the image, as well as the spatial association between nearby localized pixels.

The prognostic significance of radiomic features from MRI has been explored in cases of various malignancies, including HCC, breast cancer, nasopharyngeal carcinoma, and pancreatic cancer (24–26). However, associating a single radiomic feature with complex tumor bioprocesses remains challenging. Consequently, multifactor panels are commonly constructed to estimate the outcomes of malignancies in the radiomic setting. For instance, Zhang et al. (10) developed a radiomics model combining T1WI, T2WI, and gadoxetic acid-enhanced sequences (AP, PVP, and HBP) for ER prediction in HCC patients, with the training set AUC of 0.754 and the internal validation set AUC of 0.728. Similarly, Zhao et al. (27) validated radiomics models with different sequence combinations to predict ER, with the best model (in-phase T1WI, out-phase T1WI, T2WI, AP, VP, and DP) attaining AUCs 0.831 and 0.771 in the training and validation sets, respectively. Although promising, these radiomics models do not include comparisons with functional MRI sequences. Importantly, some studies suggest that quantitative parameters of functional MRI, such as the average apparent kurtosis coefficient of DKI and the actual diffusion coefficient of IVIM, can effectively predict ER in HCC (28–30). The R2* map, a functional MRI, is widely utilized in the diagnosis and differential diagnosis of neurological diseases (31). The R2* map is currently applicable for assessing abdominal tumors, including the diagnosis of prostate cancer (32), differential diagnosis of ovarian tumors (15), and identification of the etiology of ovarian cysts (33). The R2* map has also been used to evaluate liver fibrosis (34) and to identify benign and malignant liver tumors (23). By reflecting the oxygen content of local tissue, the R2* map non-invasively indicates the tissue oxygenation levels; an increase in R2* value indicates a decrease in local tissue oxygenation capacity (35). In the present study, we established and verified for the first time an R2* map radiomics method for individualized predicting of ER in HCC patients after hepatectomy. The R2* map provides tumor heterogeneity information based on blood oxygen levels and does not require contrast injection, making it a genuinely non-invasive test.

Furthermore, the results of this study indicate that serum AFP level, and MVI can independently predict ER. Elevated AFP, a crucial HCC tumor marker, correlates with ER and is positively associated with low differentiation, MVI, and tumor recurrence in HCC patients (36, 37). We found that NLR and PLR are associated with ER in HCC, suggesting a potential connection between changes in these inflammatory markers and proinflammatory mediators influencing oncogenic effects, thereby accelerating proliferation and invasion of tumor cells (38). Despite well-established epidemiological evidence linking inflammation to cancer risk (37, 38), the underlying mechanisms remain unclear. This study reaffirmed a robust association between MVI and ER, as observed in previous studies (39–41), affirming the aggressive nature of HCC and its adverse impact on survival outcomes.

This study was not without limitations. The retrospective design, focusing only on solitary HCCs, may have introduced selection bias restricting the generalizability of the proposed model to multiple tumors. Future research should explore the link between radiomic features and ER in a broader range of tumors. Additionally, the small sample size may have compromised the model’s robustness, necessitating further optimization through large-scale, multi-center studies. The time- and labor-intensive nature of three-dimensional ROI segmentation calls for more convenient tools for automatic segmentation, which would enhance the application of radiomics in regular radiology practice. Lastly, while nomograms are widely used for summarizing prediction models, they represent only static models, require user-dependent decisions, and lack reporting standards. Web applications offer a dynamic and instantly deployable prediction tool, mitigating several of these limitations inherent to nomograms.

## 5 Conclusions

We constructed and validated a nomogram incorporating Rad-score, MVI, and serum AFP level indicators to accurately predict the ER of a singular HCC. This nomogram is demonstrated as a precise and easy to interpret tool in clinical practice, offering valuable assistance in risk stratification. Following further validation, it has the potential to guide individualized monitoring and to inform therapeutic decision-making among both clinicians and patients.

## Data availability statement

The original contributions presented in the study are included in the article/supplementary material. Further inquiries can be directed to the corresponding author.

## Ethics statement

The studies involving human participants were reviewed and approved by The Ethics Review Board of the center hospital of Zhanjiang. Written informed consent for participation was not required for this study in accordance with the national legislation and the institutional requirements.

## Author contributions

YZ: Writing – review & editing. JL: Data curation, Software, Writing – original draft. YM: Data curation, Methodology, Validation, Writing – original draft. CY: Data curation, Methodology, Software, Writing – review & editing. GQ: Data curation, Funding acquisition, Writing – review & editing. JC: Data curation, Formal analysis, Software, Writing – review & editing. XT: Data curation, Formal analysis, Software, Writing – review & editing.
